# Leveraging early HIV diagnosis and treatment in Thailand to conduct HIV cure research

**DOI:** 10.1186/s12981-019-0240-4

**Published:** 2019-09-06

**Authors:** Camilla Muccini, Trevor A. Crowell, Eugène Kroon, Carlo Sacdalan, Reshmie Ramautarsing, Pich Seekaew, Praphan Phanuphak, Jintanat Ananworanich, Donn J. Colby, Nittaya Phanuphak

**Affiliations:** 1 Vita-Salute San Raffaele University, via Stamira D’Ancona 20, 20127 Milan, Italy; 2 U.S. Military HIV Research Program, Walter Reed Army Institute of Research, Silver Spring, USA; 3 Henry M. Jackson Foundation for the Advancement of Military Medicine, Bethesda, USA; 4 SEARCH, Thai Red Cross AIDS Research Centre, Bangkok, Thailand; 5 PREVENTION, Thai Red Cross AIDS Research Centre, Bangkok, Thailand; 6 Faculty of Medicine, Chulalongkorn University, Bangkok, Thailand; 7 Department of Global Health, The University of Amsterdam, Amsterdam, The Netherlands

**Keywords:** Acquired immunodeficiency syndrome, AIDS vaccines, HIV antibodies, HIV seropositivity, AIDS serodiagnosis, Anti-retroviral agents, Highly active antiretroviral therapy, Disease reservoirs

## Abstract

Thailand has the highest prevalence of HIV among countries in Asia but has also been a pioneer in HIV prevention and treatment efforts in the region, reducing the incidence of new infections significantly over the last two decades. Building upon this remarkable history, Thailand has set an ambitious goal to stop the AIDS epidemic in the country by 2030. A key component of the strategy to achieve this goal includes scale-up of HIV screening programs to facilitate early HIV diagnosis and investment in mechanisms to support immediate initiation of antiretroviral therapy (ART). Initiation of ART during early or acute HIV infection not only reduces viremia, thereby halting onward transmission of HIV, but also may facilitate HIV remission by reducing the size of the latent HIV reservoir and preserving immune function. In Thailand, many efforts have been made to reduce the time from HIV infection to diagnosis and from diagnosis to treatment, especially among men who have sex with men and transgender women. Successfully identifying and initiating ART in individuals with acute HIV infection has been leveraged to conduct groundbreaking studies of novel strategies to achieve HIV remission, including studies of broadly-neutralizing HIV-specific monoclonal antibodies and candidate therapeutic vaccines. These efforts have mostly been deployed in Bangkok and future efforts should include other urban and more rural areas. Continued progress in HIV prevention, screening, and treatment will position Thailand to substantially limit new infections and may pave the way for an HIV cure.

## Introduction

In Thailand, there are an estimated 440,000 people living with HIV (PLWH) and 15,000 die of AIDS-related illnesses annually [1]. Despite having the highest prevalence of HIV in Asia, Thailand has gained fame for tremendously effective deployment of HIV prevention programs that reduced the number of annual new HIV infections from 115,000 in 1992 to 6400 in 2016 [2–4]. These public health interventions were most successful in decreasing HIV transmission among reproductive age adults populations and people who inject drugs (PWID) [2, 5].

Building upon this success, Thailand has set the ambitious goal of stopping AIDS by 2030 [6]. As part of the strategy to achieve this goal, the country intends to increase HIV testing coverage for key populations, including men who have sex with men (MSM), transgender people, PWID and sex workers. PLWH identified via this expanded screening will be referred immediately for antiretroviral therapy (ART) to decrease HIV transmission, improve clinical outcomes and achieve rapid viral suppression. Since 2014, the Thailand HIV guidelines have recommended ART initiation as soon as possible, regardless of CD4 cell count [7], forerunning similar recommendations from the World Health Organization (WHO) [8]. The same national guidelines also recommended pre-exposure prophylaxis (PrEP) as part of combination HIV prevention packages for people who are HIV-uninfected and at high-risk of HIV acquisition.

Thailand is moving towards a concrete realization of early diagnosis and treatment, which plays a role not only in the prevention of onward transmission of HIV, but also in minimizing the size of the HIV reservoir and preserving immune function [9, 10]. This progress in management of HIV infection affirms Thailand as a key country in developing and implementing potential strategies to achieve an HIV cure [11].

### Size and effectiveness of HIV screening programs

In order to diagnose HIV soon after the infection is acquired, screening programs must be available to populations at the highest risk of infection. In Asia, nearly 65% of new infections occur in MSM, clients of sex workers and other sexual partners of key populations [12]. However, HIV screening programs among MSM are still scarce in Asia and there is a low rate of regular HIV testing [13–15]. According to recent data from Thailand, HIV testing coverage, defined as receipt of a test in the last 12 months, was only 29% among MSM, compared with 58% among female sex workers and 61% among PWID [16]. Compared to data from 2008 to 2009, testing coverage remained stable in PWID (59.7%) and had increased in both MSM and sex workers (21.3% and 35.2%, respectively) [17]. Prevention programs are more extensive and effective in the capital city, Bangkok, than in the rest of Thailand [18, 19]. Persistent obstacles to HIV screening include people’s inability to self-identify or admit HIV risk, HIV-related stigma, and concern about side effects of ART [11, 20].

Thailand has successfully piloted a number of strategies to increase access to HIV testing for key populations, including MSM. HIV prevalence in MSM is almost ten times higher than in reproductive age adults nationwide [3] and is a staggering 28.6% in Bangkok [21]. Incentives to motivate key populations to access HIV testing include providing free tests and rapid results. Delayed test results have been linked to increased loss to follow-up after testing [22]. HIV rapid diagnostic testing can be conducted by well-trained lay providers with test accuracy similar to those performed by healthcare professionals, which is an implementation strategy that could make screening programs more widespread and improve HIV testing coverage [23].

Lay providers, who themselves may be members of or work closely with key populations, can design and deliver health services that are well tailored and responsive to the specific needs of key populations with non-discrimination and quality standards. In 2016, these key population lay providers under the key population-led health services (KPLHS) model contributed to 42% of all HIV testing and 35% of all HIV diagnoses made among MSM and transgender women (TGW) in Thailand [22].

Mobile clinics and peer-led HIV testing services have been shown to increase both access to HIV screening and HIV knowledge, especially among younger participants [24]. In addition, advances in technology such as online HIV testing services help with both the quantity and quality of HIV screening for at-risk MSM and TGW [25, 26]. Other strategies to encourage uptake of HIV prevention interventions could include provision of ancillary services such as screening for other sexually transmitted infections [22], offering self-testing as an adjunct or alternative to clinic-based HIV screening [26] and guaranteeing access to gender affirmative hormone treatment among TGW [27].

### HIV PrEP uptake among key populations

Thailand has included PrEP in its national HIV guidelines as a prevention method for people at high risk of infection since 2014 [7]. A number of programs have since then made PrEP available to key populations, including MSM, TGW, and individuals in serodiscordant couples. PrEP-30, launched in Thai Red Cross Anonymous Clinic in December 2014, was the first available PrEP service in Thailand, providing nonsubsidized PrEP for a fee of 30 THB ($1 USD) per day [28]. In November 2016, the Thai Ministry of Public Health launched PrEP2START, a public health capacity-building program that provides free PrEP to anyone at risk in eight provinces [29]. The Princess PrEP Program, supported by the Princess Soamsawali HIV Prevention fund at The Thai Red Cross AIDS Research Center, was the first key population-led PrEP program for MSM, transgender populations, sex workers, and people who use drugs, providing PrEP through eight community-based clinics in four provinces [30]. By the end of 2018, there were over 6000 people in Thailand accessing PrEP [31], and the Thai National Health Security Office announced that PrEP would be made available through the national health insurance system as early as the end of 2019 [32, 33].

### Decreasing time from diagnosis to ART initiation

Once HIV infection occurs, proviral HIV DNA rapidly integrates into resting and memory CD4 cells, where it remains transcriptionally silent [34]. This latent reservoir represents the major barrier to HIV cure. Starting ART during acute HIV infection (AHI) substantially reduces the HIV reservoir as compared to ART initiation during chronic HIV infection [9, 10]. A study conducted in Pattaya and Bangkok demonstrated that ART initiation within 5 days of HIV infection increased the likelihood of having no detectable HIV DNA in central memory CD4 cells [35]. Therefore, shortening the time between diagnosis and ART initiation may facilitate HIV cure when combined with other novel interventions.

The test-and-treat approach combines periodic HIV testing and immediate ART initiation. At-risk populations screened within this intervention strategy are motivated to initiate ART as soon as possible, thereby achieving a life expectancy similar to HIV-uninfected people [22, 36, 37]. In 2012, 810 Thai MSM and TGW were enrolled in a test-and-treat study in Bangkok, Ubon Ratchathani, Lampang and Mahasarakam that newly-diagnosed 134 (16.5%) PLWH [38]. Immediate ART initiation was recommended to all participants diagnosed with HIV and the acceptance rate was 83% [38].

Between 2015 and 2016 another test-and-treat study was conducted at five hospitals that served MSM and TGW in four Thai provinces [39]. Many participants received HIV testing for the first time, revealing that a previously-unreached key population was being screened [40]. Among those with incident HIV infection, 86.1% initiated ART and 58.6% of ART initiators did so within 2 weeks of diagnosis [39]. Although ART uptake in this program was higher than had been reported from other areas in Thailand [41], it still fell short of the UNAIDS 90-90-90 target.

At the Thai Red Cross AIDS Research Centre (TRCARC) in Bangkok, physicians offer same-day ART to participants who fulfill eligibility criteria, such as the exclusion of active tuberculosis, cryptococcal meningitis and other opportunistic infections. Same-day ART was accepted and initiated by 89.5% of 3443 individuals with newly-diagnosed HIV between July 2017 and April 2019 [42]. Successes in deploying test-and-treat programs in Thailand’s large cities need to be emulated in other parts of the country to fully realize the potential of this HIV prevention strategy.

### Diagnosis and treatment during acute HIV infection

AHI is defined as the first few weeks after HIV transmission, before the HIV antibody response has fully developed. AHI can be diagnosed by the presence of HIV RNA in the blood in the absence of HIV antibodies or by the detection of HIV antibodies by sensitive third or fourth generation antibody tests while less-sensitive second generation antibody tests and Western blot remain nonreactive or indeterminate [43]. The use of newer generation HIV test kits that detect both HIV antigens and antibodies has increased sensitivity to detect the earliest phases of HIV infection [44]. In one study in Thailand, addition of nucleic acid testing to to an HIV screening algorithm based on the 4th generation enzyme immunoassay raised the number of AHI diagnosis from 12 to 17 per 10,000 samples tested [45].

Since 2009, the Anonymous Clinic at the TRCARC has screened for AHI in over 300,000 people presenting for voluntary HIV testing and over 600 have enrolled for immediate ART and longitudinal follow-up in the RV254/SEARCH010 cohort (NCT00796146). The median duration since estimated HIV exposure was 19 (range: 3–61) days and 99% initiated ART within 1 week of AHI diagnosis [46].

ART initiation during AHI, has been shown to limit the size of the HIV reservoir and to preserve immune function [47]. Studies conducted in the RV254/SEARCH010 cohort have highlighted other benefits of early treatment, such as the potential to prevent or limit gut inflammation [48] and neurological impairment [49].

### Viral suppression rates on ART

PLWH who are virally-suppressed on ART experience improved clinical outcomes as compared to viremic individuals and cannot transmit HIV. For these reasons, achieving viral suppression is a cornerstone of HIV management and the third “90” in the UNAIDS 90-90-90 targets to end AIDS. Hoenigl et al. showed that viral suppression was rapid after early ART initiation in either AHI or chronic HIV infection, with median time to undetectable viremia being 12 weeks (interquartile range, IQR: 4–24 weeks) in each group [50]. At TRCARC, PLWH who started ART on the day of HIV diagnosis were 2.2 times more likely to be virally suppressed when compared to PLWH who started ART later after diagnosis [51]. In the RV254/SEARCH010 cohort, participants who started ART during Fiebig stage I reached viral suppression in a median of 8 weeks (IQR: 4–12), while all other Fiebig stages achieved viral suppression in a median of 12 weeks (IQR: 8–16), showing a statistically significant difference in time to viral suppression that favored earlier ART initiation even within the setting of acute infection [52]. As HIV is a chronic disease, viral suppression needs to be maintained lifelong, which for most PLWH means taking daily medication with strict adherence for decades. When ART is started during chronic HIV infection, virologic failure rates have been reported to be 10–20% at 24 weeks [53, 54]. Virologic failure is less common in individuals who initiate ART during AHI, observed in only 1.1% of 264 Thai PLWH at 24 weeks [52].

Between July 2017 and April 2019, at the TRCARC in Bangkok 89.8% of HIV infected persons on ART who received viral load testing were virally suppressed [42].

These results highlight successes in achieving viral suppression in Thailand once HIV has been diagnosed, but there is room to improve. In Thailand, recent data showed that more than 95% of PLWH knew their status, 72% were on ART and 62% were virally suppressed [12]. Successful programs to promote HIV testing and earlier ART initiation need to be scaled up nationwide in order to achieve the UNAIDS 90-90-90 targets.

### HIV cure in Thailand

The ongoing RV254/SEARCH010 cohort in Bangkok has proven to be fertile ground for the development and implementation of HIV remission trials, leveraging evidence that the lower reservoir size and preserved immune function of individuals who start ART during acute infection might facilitate viral control in the absence of ART. The study has shown that execution of observational and interventional research during the period surrounding acute HIV infection is safe, feasible, and acceptable to participant populations [55, 56]. This includes research that involves invasive procedures such as lumbar puncture and lymph node biopsy [57, 58]. To date, four HIV remission clinical trials have been completed using the cohort as a source population (Tables 1 and 2).

**Table 1:** Summary of clinical HIV remission trials in the RV254/SEARCH010 cohort in Bangkok, Thailand

Study	Inclusion criteria	Number of participants	Dose and duration of investigational agent	Duration of ATI	Key findings	Ref.
RV 411	Fiebig I, on ART for ≥ 96 weeks, HIV RNA < 50 copies/mL for ≥ 48 weeks, integrated HIV DNA in PBMCs of < 10 copies/10^6^ PBMCs, and CD4 T cell of ≥ 400 cells/mm^3^	8	N/A (study of early ART only)	Up to 24 weeks	Viral rebound was observed in all participants and at a median time of 26 days. ART started in Fiebig I stage did not prevent or delay viral rebound	[59]
RV 397	Fiebig I–III, on ART for ≥ 96 weeks, HIV RNA < 50 copies/mL for ≥ 48 weeks, integrated HIV DNA in PBMCs of < 10 copies/10^6^ PBMCs, and CD4 T cell of ≥ 400 cells/mm^3^	18	VRC01 40 mg/kg IV every 3 weeks for up to 24 weeks	Up to 48 weeks	VRC01 was generally safe and was associated with a trend toward delayed viral rebound: the placebo group experienced viral rebound at a median of 14 days, whereas participants in the VRC01 group at a median of 26 days	[60]
RV 409	Fiebig III–IV, on ART for ≥ 42 weeks, HIV RNA < 50 copies/mL for ≥ 28 weeks, CD4 T cell of ≥ 450 cells/mm^3^	15	Vorinostat PO 400 mg/day 14 days on/off (3 cycles), hydroxychloroquine PO 200 mg BID, maraviroc PO 600 mg BID for 10 weeks	Up to 24 weeks	VHM was well tolerated in the majority of participants. No changes in total HIV DNA in PBMCs were described. All Fiebig III/IV treated participants had viral rebound after ATI; median time to viral rebound was 22 days	[61]
RV 405	Fiebig I–IV, on ART for ≥ 4 weeks, HIV RNA < 50 copies/mL for ≥ 48 weeks, CD4 T cell of ≥ 400 cells/mm^3^	26	Ad26 vaccine 5 * 10^10^ viral particle per 0.5 mL IM at week 0 and 12, MVA vaccine 10^8^ plaque-forming unit per 0.5 mL IM at week 24 and 48	Up to 36 weeks	Ad26/MVA was well-tolerated and it contributed to a modest delay in time to viral rebound after analytic treatment interruption	[62]

*pts.* participants, *ref.* reference, *ART* antiretroviral therapy, *PBMCs* peripheral blood mononuclear cells, *IV* intravenous, *PO* per os, *Ad26* adenovirus type 26 vector prime, *IM* intramuscular, *MVA* modified vaccinia Ankara

**Table 2:** Participant characteristics in HIV cure trials

Study agents	RV411	RV397		RV409		RV405	
	N/A	VRC01	Placebo	VHM	Placebo	Ad26/MVA	Placebo
Number of participants	8	13	5	10	5	17	9
Median age	29	32	25	28	26	24	25
Male (%)	87.5	100	100	89	75	100	100
Median HIV RNA pre-ART, log_10_ c/mL	4.3	3.1	2.7	6.1	5.6	5.9	6.4
Median CD4, cells/mm^3^ pre-ART	413	769	552	397	532	633	586
Median CD4/CD8 ratio pre-ART	0.8	1.1	0.9	0.4	0.8	0.6	0.6
Median duration of ART (years)	2.8	3.1	2.7	4.3	3.0	2.2	2.2
Fiebig stage (number)	I: 8	I/II: 8 III: 5	I/II: 3 III: 2	III: 8 IV: 2	III: 5	I: 0 II: 6 III: 6 IV: 5	I: 1 II: 4 III: 4 IV: 0

*VHM* vorinostat, hydroxychloroquine, maraviroc, *Ad26* adenovirus type 26 vector prime, *MVA* modified vaccinia Ankara, *ART* antiretroviral therapy

RV411 was a study of analytic treatment interruption (ATI) in 8 participants who started ART during the earliest stage of AHI (Fiebig I) and were treated for a median of 2.8 years. Following ATI, all participants experienced viral rebound above 20 copies/mL at a median of 26 (range 13–48) days. This single-arm study demonstrated that very early ART alone was not sufficient to control or eradicate HIV [59].

RV397 was a randomized, placebo-controlled clinical trial evaluating the safety and efficacy of a broadly neutralizing human monoclonal antibody (VRC01) targeted against the HIV CD4 binding site in 18 adults who initiated ART during AHI [60]. Participants were closely monitored and restarted ART when plasma HIV RNA was above 1000 copies/mL on two separate measurements. VRC01 modestly delayed the time to viral rebound, which occurred at a median of 14 days after ATI in the placebo group and 26 days after ATI in the VRC01 group (p = 0.051). One VRC01 recipient maintained undetectable peripheral HIV RNA through week 42. This randomized study demonstrated that VRC01 monotherapy was insufficient to maintain viral suppression in most individuals, even in this carefully selected population [60].

In a test of the “kick and kill” strategy, 15 acutely-treated participants were randomized to receive either ART alone or in combination with vorinostat (a latency reversal agent), maraviroc (an entry inhibitor), and hydroxychloroquine (an immune modulator) [61]. At week 10 all medications were stopped, and ATI was begun. Time to viral rebound > 1000 copies/mL, which occurred at a median of 22 days, did not differ significantly between the intervention and placebo arms. No changes were observed both in total HIV DNA in peripheral blood mononuclear cells (PBMCs), in T cell and soluble immune activation markers. Furthermore, ART duration, total HIV DNA in PBMCs, single copy HIV RNA and CD4/CD8 ratio did not predict time to viral load.

RV405 was a randomized, placebo-controlled study of a therapeutic vaccine using an Adenovirus type 26 vector prime and modified vaccinia Ankara boost combination with mosaic inserts in HIV-infected adults who initiated ART during AHI. A total of 26 participants were enrolled in the active vaccine (n = 17) and placebo (n = 9) arms. As in all ATI studies, participants were monitored frequently, and ART was reinitiated when viral rebound was detected [62]. The study showed that the vaccine regimen was safe, well-tolerated, and induced a robust immunologic response; but that it resulted in only a slight delay in time to viral rebound after ATI. Future trials may investigate therapeutic vaccine regimens with the addition of immunomodulators and different immunogens.

## Conclusions

Thailand has emerged as a pioneer in efforts to prevent, treat, and ultimately cure HIV with an ambitious national strategy to stop the AIDS epidemic in the country by 2030. Successful pilot studies of expanded HIV screening and test-and-treat programs now need to be scaled up to reach diverse populations throughout the country, including key underserved populations such as MSM, TGW, and PWID. Thailand has been uniquely successful in providing prevention modalities and initiating ART in individuals with acute HIV infection, yielding a valuable source population for the conduct of groundbreaking studies of novel strategies to achieve HIV remission. Thailand is poised to continue to have a leading role in HIV prevention and cure research thanks to the combined efforts from communities of key and affected populations, researchers, government and policy makers.

## Data Availability

Data sharing is not applicable to this article as no datasets were generated or analysed during the conduct of this review.

## Abbreviations

AHI: acute HIV infection
AIDS: acquired immune deficiency syndrome
ART: antiretroviral therapy
ATI: analytic treatment
IQR: interquartile range
KPLHS: key population-led health services
MSM: men who have sex with men
O2O: Online-to-Offline
PBMC: peripheral blood mononuclear cell
PLWH: people living with HIV
PrEP: pre-exposure prophylaxis
PWID: people who inject drugs
TGW: transgender women
TRCARC: Thai Red Cross AIDS Research Centre
UNAIDS: the Joint United Nations Programme on HIV/AIDS interruption
WHO: World Health Organization
